# Normal CFTR Inhibits Epidermal Growth Factor Receptor-Dependent Pro-Inflammatory Chemokine Production in Human Airway Epithelial Cells

**DOI:** 10.1371/journal.pone.0072981

**Published:** 2013-08-16

**Authors:** Suil Kim, Brittney A. Beyer, Courtney Lewis, Jay A. Nadel

**Affiliations:** 1 Division of Pulmonary and Critical Care Medicine, Department of Medicine, Portland Veterans Affairs Medical Center, Oregon Health & Science University, Portland, Oregon, United States of America; 2 Department of Physiology and Pharmacology, Oregon Health & Science University, Portland, Oregon, United States of America; 3 Cardiovascular Research Institute, University of California San Francisco, San Francisco, California, United States of America; 4 Department of Medicine, University of California San Francisco, San Francisco, California, United States of America; 5 Department of Physiology, University of California San Francisco, San Francisco, California, United States of America; University of North Carolina at Chapel Hill, United States of America

## Abstract

Mutations in cystic fibrosis transmembrane conductance regulator (CFTR) protein cause cystic fibrosis, a disease characterized by exaggerated airway epithelial production of the neutrophil chemokine interleukin (IL)-8, which results in exuberant neutrophilic inflammation. Because activation of an epidermal growth factor receptor (EGFR) signaling cascade induces airway epithelial IL-8 production, we hypothesized that normal CFTR suppresses EGFR-dependent IL-8 production and that loss of CFTR at the surface exaggerates IL-8 production via activation of a pro-inflammatory EGFR cascade. We examined this hypothesis in human airway epithelial (NCI-H292) cells and in normal human bronchial epithelial (NHBE) cells containing normal CFTR treated with a CFTR-selective inhibitor (CFTR-172), and in human airway epithelial (IB3) cells containing mutant CFTR versus isogenic (C38) cells containing wild-type CFTR. In NCI-H292 cells, CFTR-172 induced IL-8 production EGFR-dependently. Pretreatment with an EGFR neutralizing antibody or the metalloprotease TACE inhibitor TAPI-1, or TACE siRNA knockdown prevented CFTR-172-induced EGFR phosphorylation (EGFR-P) and IL-8 production, implicating TACE-dependent EGFR pro-ligand cleavage in these responses. Pretreatment with neutralizing antibodies to IL-1R or to IL-1alpha, but not to IL-1beta, markedly suppressed CFTR-172-induced EGFR-P and IL-8 production, suggesting that binding of IL-1alpha to IL-1R stimulates a TACE-EGFR-IL-8 cascade. Similarly, in NHBE cells, CFTR-172 increased IL-8 production EGFR-, TACE-, and IL-1alpha/IL-1R-dependently. In IB3 cells, constitutive IL-8 production was markedly increased compared to C38 cells. EGFR-P was increased in IB3 cells compared to C38 cells, and exaggerated IL-8 production in the IB3 cells was EGFR-dependent. Activation of TACE and binding of IL-1alpha to IL-1R contributed to EGFR-P and IL-8 production in IB3 cells but not in C38 cells. Thus, we conclude that normal CFTR suppresses airway epithelial IL-8 production that occurs via a stimulatory EGFR cascade, and that loss of normal CFTR activity exaggerates IL-8 production via activation of a pro-inflammatory EGFR cascade.

## Introduction

The potent neutrophil chemokine interleukin (IL)-8 [1] is produced and secreted in the airways as part of innate immune responses to inhaled “invaders” (eg, bacteria, viruses, cigarette smoke). In cystic fibrosis (CF), a disease caused by mutations in the CF transmembrane conductance regulator (CFTR) protein [2], [3], exaggerated airway epithelial IL-8 production [4]–[6] leads to persistent neutrophilic inflammation, a serious and presently untreated feature of CF airway disease [7]. There is growing evidence that exaggerated IL-8 production may be an intrinsic property of airway epithelial cells lacking normal CFTR. For example, increased levels of IL-8 and neutrophils have been observed in the airways of infants with CF in the absence of detectable infection [8], and in sterile CF fetal tracheal grafts explanted under the skin of immunodeficient mice compared to non-CF controls [9]. In addition, airway epithelial cells that contain mutant CFTR have been shown to produce more IL-8 in response to bacterial products [10]–[12] and to IL-1 [13], and to produce more IL-8 in the constitutive state [10], [11], [14]–[16], than isogenic cells corrected with wild-type CFTR. Finally, treatment of airway epithelial cells that contain normal CFTR with CFTR-selective inhibitors has been shown to induce IL-8 production [17]–[19]. Together, these findings suggest that loss of normal CFTR function exaggerates airway epithelial IL-8 production.

Activation of an epidermal growth factor receptor (EGFR) signaling cascade has been implicated in airway epithelial IL-8 production [20]–[22]. The airways of healthy adult humans express EGFR and EGFR ligands only sparsely [23]. Expression of EGFR and its ligands is increased in the airways of subjects with CF [24]. Autocrine activation of an EGFR signaling cascade involves proteolytic cleavage of membrane-anchored EGFR pro-ligands at the cell surface by metalloproteases such as TNF-alpha converting enzyme (TACE; [25], [26]) and subsequent binding of the mature soluble ligand to EGFR. Subauste and Proud first showed that treatment of airway epithelial cells with EGFR ligands results in IL-8 production [22]. Since then, multiple stimuli have been shown to induce airway epithelial IL-8 production via activation of a surface TACE-EGFR cascade [20], [21], [27]–[30], suggesting that this cascade is a convergent pathway for airway epithelial IL-8 production.

Because loss of normal CFTR function and activation of a TACE-EGFR cascade both lead to airway epithelial IL-8 production, here we hypothesized that loss of normal CFTR function removes the inhibitory role of CFTR and thus exaggerates IL-8 production via activation of a pro-inflammatory TACE-EGFR cascade. We tested this hypothesis using two complementary approaches. In the first approach, the effects of a CFTR-selective inhibitor on IL-8 production were examined in airway epithelial cells that contain normal CFTR. We chose the CFTR-selective inhibitor CFTR-172 [31] because CFTR-172 has been shown to induce IL-8 production in airway epithelial cells containing normal CFTR [17]–[19], and because Perez et al. reported no off-target effects of CFTR-172 in airway epithelial cells containing mutant CFTR [17]. We chose human airway epithelial (NCI-H292) cells because they contain normal CFTR [32], and because these cells are a well characterized and widely used model system of IL-8 production [20], [29], [30]. Normal human bronchial epithelial (NHBE) cells were used to confirm findings described in the NCI-H292 cells. In the second approach, the effects of CFTR on constitutive IL-8 production were examined in airway epithelial cells containing mutant CFTR (IB3 cells; [33]) and in isogenic cells complemented with wild-type CFTR (C38 cells; [34]). We chose the IB3 and C38 cells because they have been widely used to examine the effects of CFTR on IL-8 production [10], [12], [15], [16], [35]–[37].

Consistent with our hypothesis, here we show that loss (or removal) of CFTR function exaggerates airway epithelial IL-8 production via activation of a pro-inflammatory TACE-EGFR cascade. Further, we show that binding of IL-1alpha to IL-1R activates the TACE-EGFR cascade in airway epithelial cells lacking normal CFTR function, exaggerating IL-8 production in these cells.

## Materials and Methods

### Materials

The CFTR inhibitor CFTR-172 [31] was provided by Dr. Alan Verkman at the Univ. of California San Francisco. AG1478, AG1295, TAPI-1, EGFR neutralizing antibody (Ab-3), TNFR neutralizing antibody (Ab-1), and cycloheximide were purchased from Calbiochem (LaJolla, CA). Neutralizing antibodies against IL-1R, IL-1alpha and IL-1beta were purchased from R&D Systems (Minneapolis, MN).

### Cell culture

Cells containing normal CFTR: A) Human airway epithelial (NCI-H292) cells [38], which express wild-type CFTR [32], were purchased from American Type Culture Collection (Manassas, VA), and were grown in RPMI 1640 medium containing 10% fetal bovine serum, penicillin (100 U/ml), streptomycin (100 µcg/ml), and 25 mM HEPES at 37°C in a humidified 5% CO2 water-jacketed incubator as described previously [30]. NCI-H292 cells have been shown to contain the intermediate filament protein keratin [38], confirming the epithelial origin of these cells. Because cell lines such as NCI-H292 show variability in their responses to stimuli and inhibitors at different passages, all experiments were performed with cells from passages 80–90. B) Normal human bronchial epithelial (NHBE) cells were purchased from Lonza (Walkersville, MD) or were isolated from human tracheas provided by the Pacific Northwest Transplant Bank as described previously [39]. In brief, tracheas were incubated in Ca^+2^- and Mg^+2^-containing DMEM supplemented with 0.5% pronase, penicillin (100 U/ml), streptomycin (100 mcg/ml), and amphotericin B (0.25 mcg/ml) overnight at 4°C. After overnight incubation, airway epithelial cells were detached from the stroma by gentle agitation, digestion was stopped by the addition of fetal bovine serum to a final concentration of 20%, and the cells were collected by centrifugation at 300 g for 10 min. NHBE cells were grown in immersed culture in bronchial epithelial growth medium (BEGM; Lonza) as described previously [30]. NHBE cells were grown in immersed culture instead of air-liquid interface to maintain culture conditions similar to those for the NCI-H292 cells. NHBE cells have been shown to produce IL-8 in immersed culture [30]. Experiments with NHBE cells were performed with passages 2–4 to limit variable responses.

Cells containing mutant CFTR versus isogenic cells corrected with wild-type CFTR: Human airway epithelial (IB3) cells expressing mutant CFTR (deltaF508/W1282X; [33]) and isogenic airway epithelial (C38) cells complemented with wild-type CFTR [34] were purchased from American Type Culture Collection, and were grown in LHC-8 medium (Invitrogen, Grand Island, NY) containing 5% fetal bovine serum, penicillin (100 U/ml), and streptomycin (100 mcg/ml) at 37°C in a humidified 5% CO2 water-jacketed incubator. Experiments with the IB3 and C38 cells were performed with passages 5–15 to limit variable responses.

In experiments utilizing NCI-H292 or NHBE cells, confluent cultures were serum-starved (NCI-H292) or incubated with EGF-free BEGM (NHBE) for 2 h before the addition of CFTR-172. Chemical inhibitors and neutralizing antibodies were added 30 min before (“0 h”) or at various times after stimulation with CFTR-172. Cell lysates and supernatants were harvested at various times up to 24 h after stimulation for measurement of IL-8, EGFR-P, and IL-1alpha. In experiments utilizing the IB3 and C38 cells, confluent cultures were washed and incubated with serum-free medium in the presence or absence of chemical inhibitors and neutralizing antibodies. Cell lysates and supernatants were harvested at various times up to 24 h for measurement of IL-8 and at 1 h for measurement of EGFR-P and IL-1alpha. The 1 h time point was chosen for measurement of EGFR-P and IL-1alpha because EGFR-P was maximal at this time point (data not shown) and because IL-1alpha binding to IL-1R contributed to EGFR-P at 1 h (see Results).

### siRNA preparation and transfection of cells

TACE siRNA knockdown and confirmation of specific TACE silencing were performed as described previously [26]. In brief, subconfluent (approximately 50%) NCI-H292 cells, IB3 cells, or C38 cells were transfected with TACE siRNAs (SMARTpool L-003453; Dharmacon, Lafayette, CO), or non-targeting control siRNA (Non-targeting pool D-001810; Dharmacon) using Lipofectamine 2000 (Invitrogen, Carlsbad, CA) according to the manufacturer’s instructions.

### Measurement of IL-8, EGFR-P, and IL-1alpha

IL-8 in cell culture supernatants, EGFR-P in cell lysates, and IL-1alpha in cell culture supernatants were measured by sandwich ELISA kits according to the manufacturer’s instructions (DuoSet IC; R&D Systems). IL-8, EGFR-P and IL-1alpha results were expressed as pg of product per mcg of total protein in the cell lysate.

### Immunoblotting

To examine TACE, lysates from NCI-H292, IB3, or C38 cells were prepared and equal amounts of protein were separated by 7.5% SDS-PAGE, transferred to polyvinylidene difluoride membranes (Biorad, Hercules, CA), and blotted with a mouse monoclonal antibody against human TACE (Santa Cruz Biotechnology, Santa Cruz, CA). Bound antibody was visualized with enhanced chemiluminescence (Amersham, Piscataway, NJ). Immunoblots for TACE were scanned, and band intensities were quantified with NIH Image 1.63 software (developed at the National Institutes of Health and available for free download at rsbweb.nih.gov).

### Statistical analysis

All data are expressed as means ± standard error (SE). One-way ANOVA and unpaired Student’s *t* test were used to determine statistically significant differences between groups (P<0.05 for the null hypothesis).

## Results

### Studies in airway epithelial cells containing normal CFTR

#### CFTR-172 induces IL-8 production in NCI-H292 cells EGFR- and TACE-dependently

**Figure 1:**
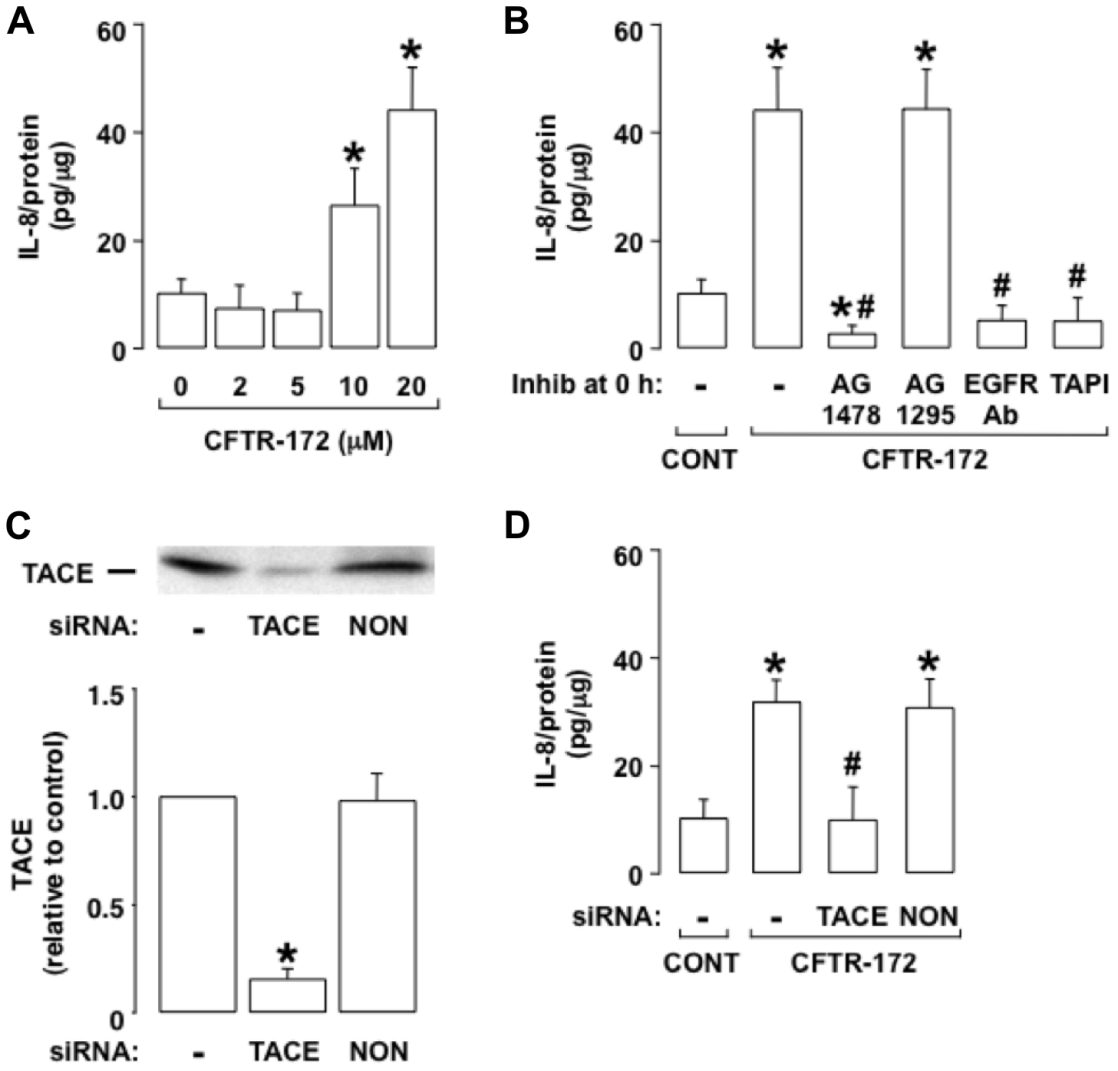
CFTR-172-induced IL-8 production involves EGFR and TACE. **A.** IL-8 was measured in NCI-H292 cells incubated for 24 h with medium alone (0 mcM CFTR-172; control) or with various concentrations of CFTR-172 (2, 5, 10, and 20 mcM). **B.** IL-8 was measured in NCI-H292 cells incubated for 24 h with medium alone (CONT) or with added CFTR-172 (20 mcM). Inhibitors were not added (–) or the EGFR-selective inhibitor AG1478 (10 mcM), the PDGFR-selective inhibitor AG1295 (10 mcM), an EGFR neutralizing antibody (5 mcg/ml), or the TACE inhibitor TAPI-1 (30 mcM) was added 30 min before treatment with CFTR-172. **C.** TACE was measured by immunoblot in NCI-H292 cells treated with Lipofectamine 2000 alone (-; control) or transfected with TACE siRNA (100 nM) or non-targeting siRNA (100 nM; NON). A blot representative of three independent experiments is shown. Band intensities were quantified, and the intensity of the control band was set arbitrarily to 1. **D.** IL-8 was measured in NCI-H292 cells treated with Lipofectamine 2000 alone (–) or in NCI-H292 cells transfected with TACE siRNA (100 nM) or nontargeting siRNA (100 nM; NON) and incubated for 72 h before addition of medium alone (CONT) or CFTR-172 for 24 h. **A-D.** Values are means ± SD; n = 5, except for **C** (n = 3). P<0.05 compared with control (*). P<0.05 compared with CFTR-172 alone (#).

The CFTR-selective inhibitor CFTR-172 has been reported to induce IL-8 production in airway epithelial cells that contain normal CFTR [17]–[19]. Because multiple stimuli have been shown to induce IL-8 production in airway cells via activation of an EGFR signaling pathway [20], [21], [29], [30], we hypothesized that CFTR-172-induced IL-8 production occurs EGFR-dependently. We tested this hypothesis in human airway epithelial (NCI-H292) cells, which contain normal CFTR [32]. The addition of CFTR-172 induced IL-8 production at 24 h in NCI-H292 cells dose-dependently (Fig. 1A). Pretreatment with the EGFR-selective inhibitor AG1478 prevented the IL-8 production induced by CFTR-172 completely (Fig. 1B), whereas pretreatment with the platelet-derived growth factor receptor inhibitor AG1295 had no significant effect on IL-8 production (Fig. 1B). In addition, pretreatment with an EGFR neutralizing antibody (which prevents ligand binding to EGFR) inhibited the IL-8 production induced by CFTR-172 completely (Fig. 1B). Together, these results implicate ligand-dependent EGFR activation in IL-8 production induced by CFTR-172.

Because the metalloprotease TACE has been shown to cleave EGFR pro-ligands, thus activating EGFR [25], [26], we examined whether TACE plays a role in CFTR-172-induced IL-8 production in the NCI-H292 cells. Pretreatment with TAPI-1 [40], a relatively selective inhibitor of TACE, prevented CFTR-172-induced IL-8 production completely (Fig. 1B). TACE siRNA knockdown (Fig. 1C) reproduced the inhibitory effect of TAPI-1 on IL-8 production induced by CFTR-172 (Fig. 1D), whereas transfection with a non-targeting siRNA control had no significant effect on the IL-8 response (Fig. 1D). Together, these results implicate metalloprotease TACE-dependent EGFR pro-ligand cleavage in CFTR-172-induced IL-8 production.

#### A late phase of EGFR-P induced by CFTR-172 leads to IL-8 production

**Figure 2:**
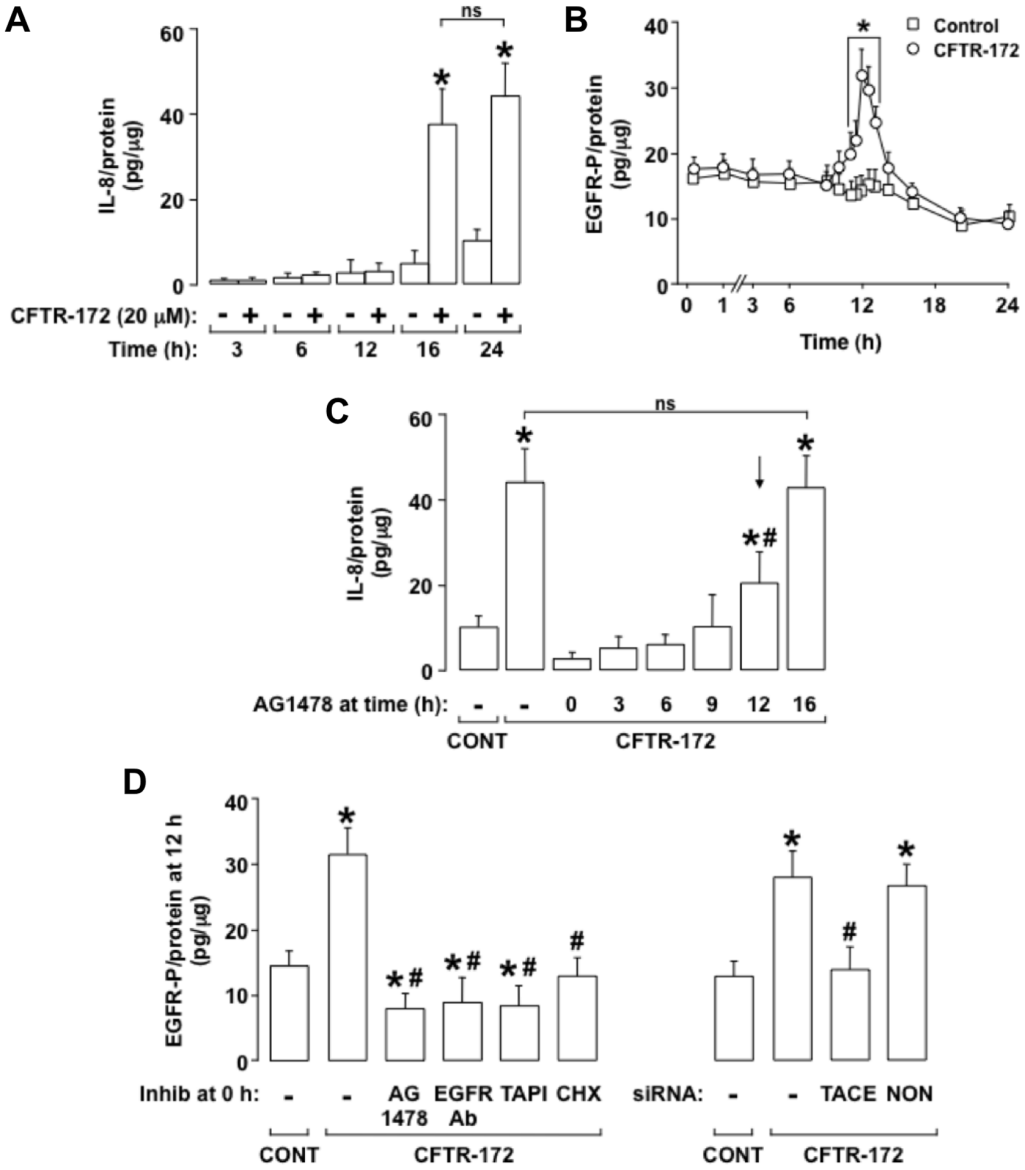
A late phase of EGFR-P leads directly to CFTR-172-induced IL-8 production. **A.** IL-8 was measured in NCI-H292 cells incubated for various times (3, 6, 12, 16, and 24 h) with medium alone (–; control) or with added CFTR-172 (+). **B.** EGFR-P was measured in NCI-H292 cells incubated with medium alone (open squares; control) or with CFTR-172 (20 mcM; open circles) starting at time 0 h for the times indicated. **C.** IL-8 was measured in NCI-H292 cells incubated for 24 h with medium alone (CONT) or with added CFTR-172 (20 mcM). The EGFR inhibitor AG1478 (10 mcM) was not added (–) or was added 0, 3, 6, 9, 12, or 16 h after CFTR-172 treatment. The arrow denotes the peak of EGFR-P at 12 h. **D.** EGFR-P was measured in NCI-H292 cells incubated for 12 h with medium alone (CONT) or with added CFTR-172 (20 mcM). Inhibitors were not added (–) or the EGFR inhibitor AG1478 (10 mcM), an EGFR neutralizing antibody (5 mcg/ml), the TACE inhibitor TAPI-1 (30 mcM), or the protein synthesis inhibitor cycloheximide (10 mcg/ml; CHX) was added 30 min before treatment with CFTR-172 (left panels). For siRNA experiments (right panels), NCI-H292 cells were treated with Lipofectamine 2000 alone (–) or were transfected with TACE siRNA (100 nM) or nontargeting siRNA (100 nM; NON) and incubated for 72 h before addition of medium alone or CFTR-172 for 12 h. **A-D.** Values are means ± SD; n = 5. P<0.05 compared with control (*). P<0.05 compared with CFTR-172 alone (#). P>0.05 (ns).

Because the production and release of IL-8 induced by CFTR-172 was delayed, occurring almost entirely between 12 and 16 h after CFTR-172 addition (Fig. 2A), we examined the effects of CFTR-172 on EGFR phosphorylation (EGFR-P) over time. CFTR-172 induced a late phase of EGFR-P that peaked at approximately 12 h and then declined to control levels by 14 h (Fig. 2B). Next, we examined the effect of this late EGFR-P on CFTR-172-induced IL-8 production. When the EGFR-selective inhibitor AG1478 was added up to 9 h after treatment with CFTR-172 (ie, before the onset of EGFR-P, see Fig. 2B), IL-8 production was inhibited completely (Fig 2C). When AG1478 was added 12 h after CFTR-172 (ie, at the peak of EGFR-P, see Fig. 2B), IL-8 production was inhibited partially (Fig. 2C). When AG1478 was added 16 h after CFTR-172 (ie, after EGFR-P had occurred, see Fig. 2B), IL-8 production was not inhibited significantly (Fig. 2C). Together, these results indicate that the late phase of EGFR-P induced by CFTR-172 leads promptly to IL-8 production.

Next we examined whether EGFR-P induced by CFTR-172 involves TACE-dependent EGFR pro-ligand cleavage. Pretreatment with AG1478, with an EGFR neutralizing antibody, or with TAPI-1, or TACE siRNA knockdown, prevented CFTR-172-induced EGFR-P completely (Fig. 2D). Further, pretreatment with the protein synthesis inhibitor cycloheximide also prevented CFTR-172-induced EGFR-P (Fig. 2D). Together, these results implicate TACE-dependent EGFR pro-ligand cleavage in CFTR-172-induced EGFR-P, and they show that this EGFR-P requires new protein synthesis.

#### IL-1alpha binding to IL-1R contributes to CFTR-172-induced EGFR-P and IL-8 production

**Figure 3:**
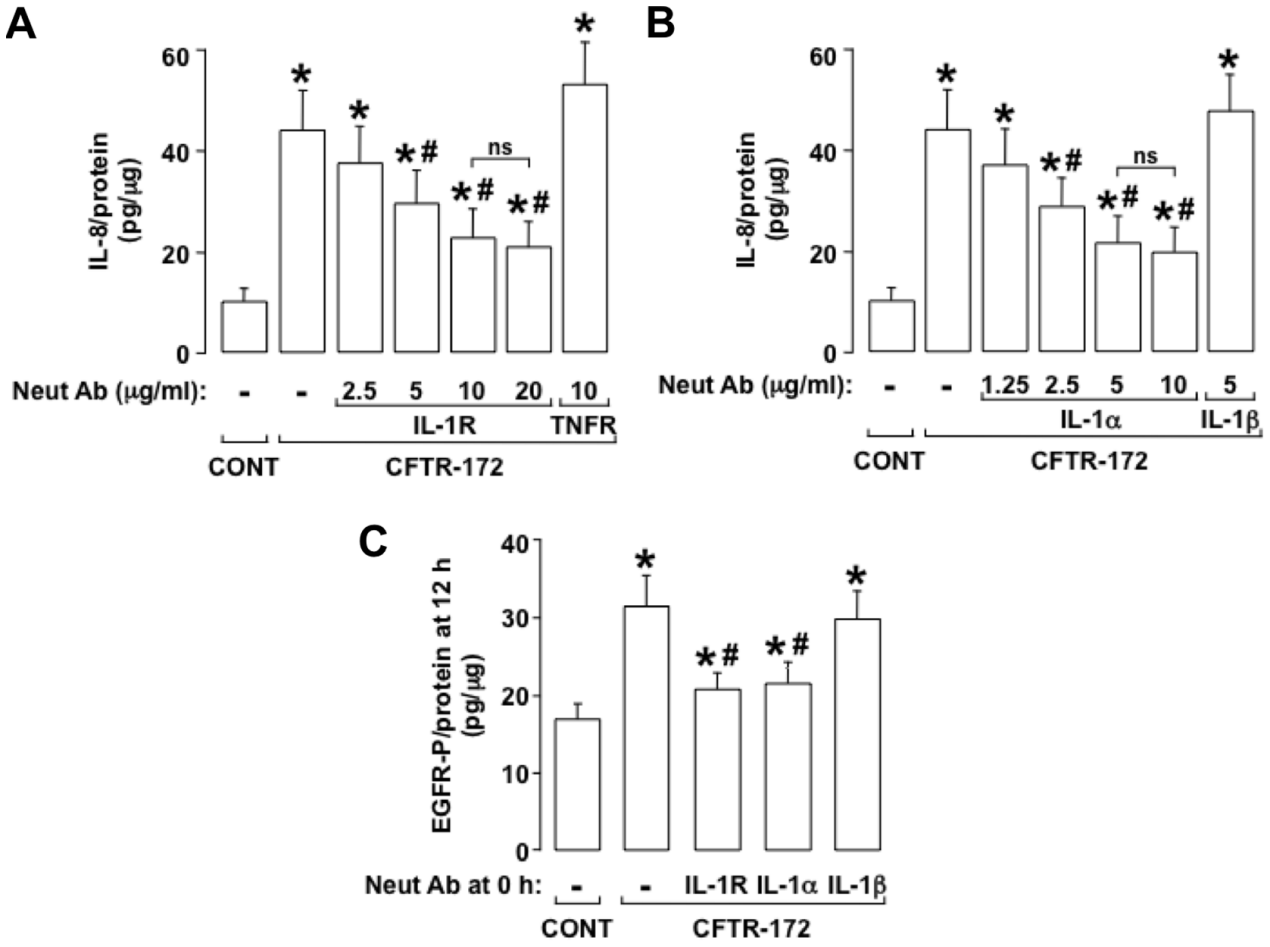
IL-1alpha binding to IL-1R contributes to IL-8 production and to EGFR-P induced by CFTR-172. **A, B.** IL-8 was measured in NCI-H292 cells incubated for 24 h with medium alone (CONT) or with added CFTR-172 (20 mcM). Neutralizing antibodies were not added (–) or (**A**) various concentrations of an IL-1R neutralizing antibody (2.5, 5, 10, and 20 mcg/ml) or a TNFR neutralizing antibody (10 mcg/ml) or (**B**) various concentrations of an IL-1alpha neutralizing antibody (1.25, 2.5, 5, and 10 mcg/ml) or an IL-1beta neutralizing antibody (5 mcg/ml) were added 30 min before CFTR-172 treatment. **C.** EGFR-P was measured in NCI-H292 cells incubated for 12 h with medium alone (CONT) or with added CFTR-172 (20 mcM). Neutralizing antibodies were not added (–) or neutralizing antibodies against IL-1R (10 mcg/ml), IL-1alpha (5 mcg/ml), or IL-1beta (5 mcg/ml) were added 30 min before treatment with CFTR-172. **A-C.** Values are means ± SD; n = 5. P<0.05 compared with control (*). P<0.05 compared with CFTR-172 alone (#). P>0.05 (ns).

Because IL-1R blockade has been shown to suppress IL-8 production in CF airway epithelial cells [41], we examined whether proteins in the IL-1R signaling pathway contribute to IL-8 production in NCI-H292 cells treated with CFTR-172. Pretreatment with an IL-1R neutralizing antibody (which prevents ligand binding to IL-1R) inhibited the IL-8 production induced by CFTR-172 markedly and dose-dependently, whereas a TNFR neutralizing antibody had no significant effect on the IL-8 response (Fig. 3A), implicating ligand binding to IL-1R in CFTR-172-induced IL-8 production. To identify the IL-1R ligand responsible for the IL-8 response to CFTR-172, we pretreated NCI-H292 cells with neutralizing antibodies to the IL-1R ligands IL-1alpha or IL-1beta. Neutralization of IL-1alpha but not IL-1beta inhibited CFTR-172-induced IL-8 production markedly and dose-dependently (Fig. 3B), implicating IL-1alpha in the IL-8 response. Pretreatment with the IL-1R or IL-1alpha neutralizing antibodies, but not with the IL-1beta neutralizing antibody, inhibited CFTR-172-induced EGFR-P markedly (Fig. 3C). Together, these results suggest that binding of IL-1alpha but not IL-1beta to IL-1R leads to CFTR-172-induced EGFR-P and to subsequent IL-8 production.

**Figure 4:**
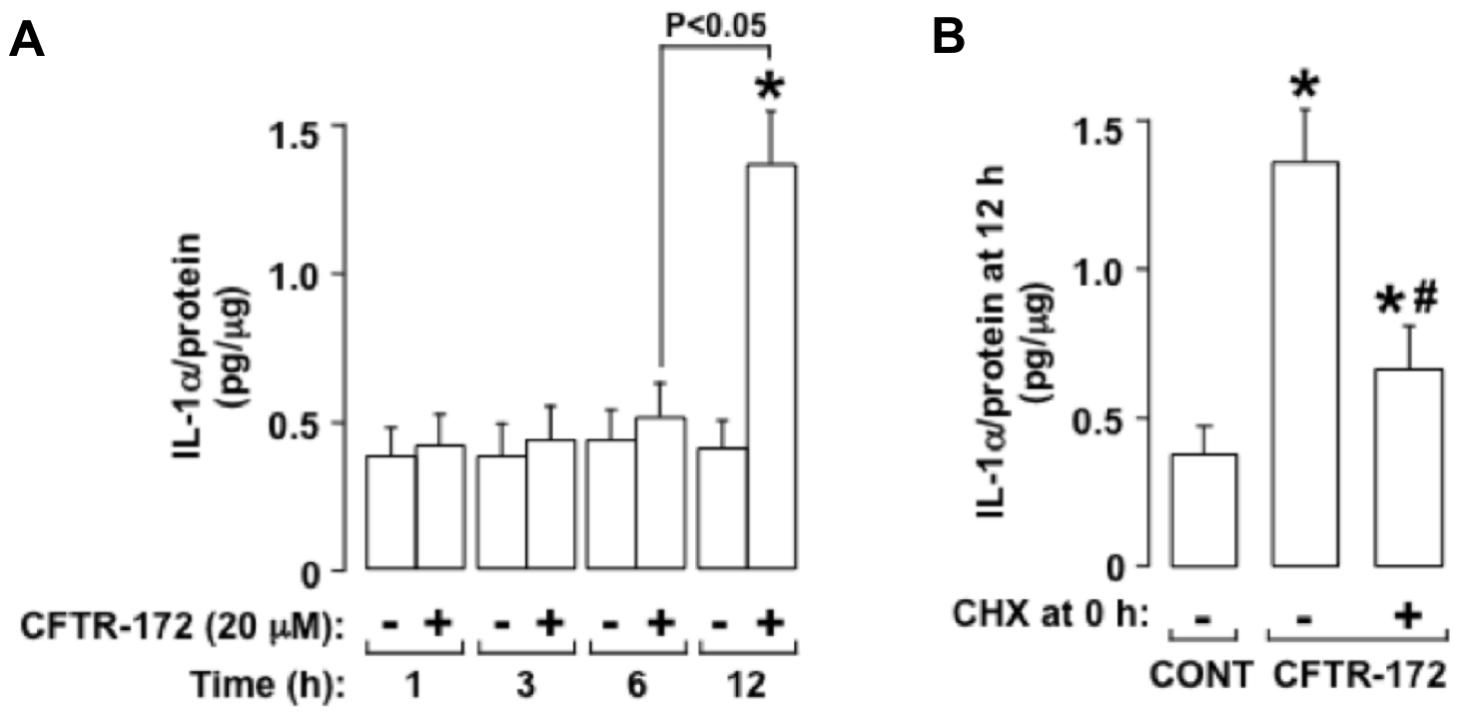
CFTR-172 induces the production and release of IL-1alpha. **A.** IL-1alpha was measured in cell supernatants of NCI-H292 cells incubated for various times (1, 3, 6, and 12 h) with medium alone (–; control) or with added CFTR-172 (+; 20 mcM). **B.** IL-1alpha was measured in cell supernatants of NCI-H292 cells incubated for 12 h with medium alone (CONT) or with added CFTR-172. Inhibitors were not added (–) or the protein synthesis inhibitor cycloheximide (10 mcg/ml; CHX) was added 30 min before treatment with CFTR-172. **A, B.** Values are means ± SD; n = 3. P<0.05 compared with control (*). P<0.05 compared with CFTR-172 alone (#).

Next we measured IL-1alpha released into the medium at various times after CFTR-172 addition. CFTR-172 had no significant effect on IL-1alpha levels at 1, 3 or 6 h, whereas at 12 h (the peak of EGFR-P, see Fig. 2B), IL-1alpha levels were increased markedly (Fig. 4A). Further, pretreatment with the protein synthesis inhibitor cycloheximide markedly suppressed the increase in IL-1alpha levels induced by CFTR-172 (Fig. 4B). Together, these results indicate that CFTR-172 induces the production and release of IL-1alpha protein.

#### CFTR-172-induced IL-8 production in NHBE cells involves EGFR, TACE, and IL-1alpha binding to IL-1R

**Figure 5:**
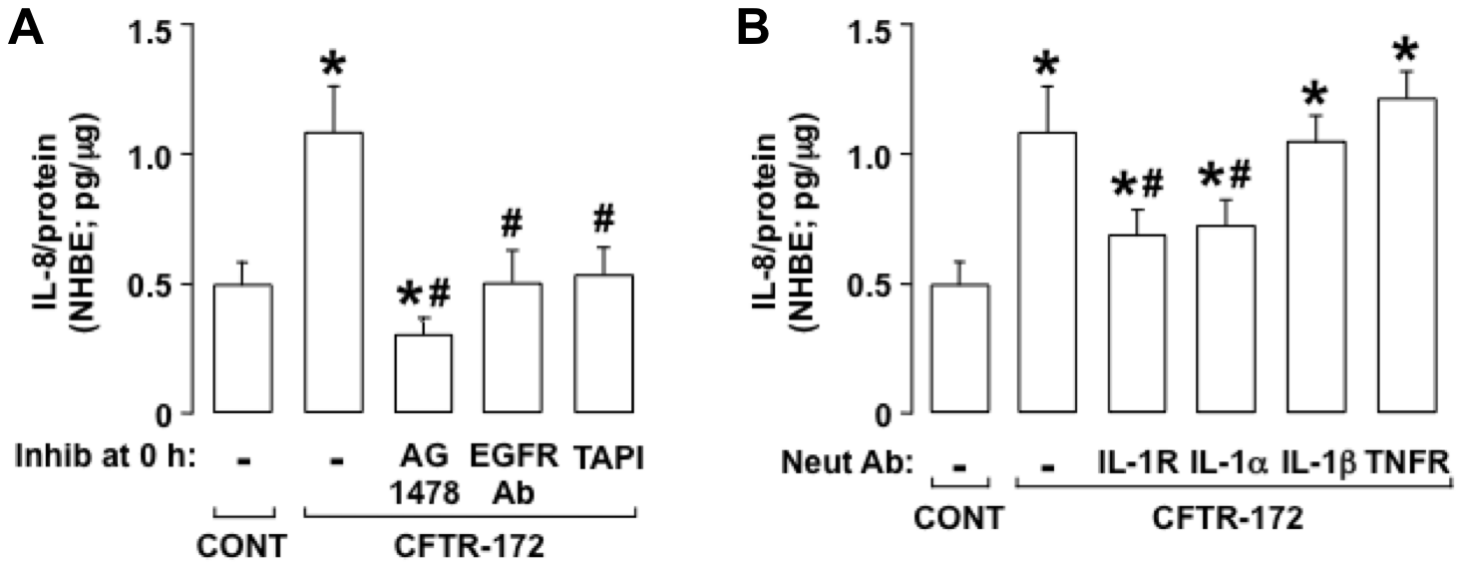
In NHBE cells, CFTR-172 induces IL-8 production via EGFR, TACE, and IL-1alpha binding to IL-1R. **A, B.** IL-8 was measured in NHBE cells incubated for 24 h with medium alone (CONT) or with added CFTR-172 (20 mcM). Inhibitors or neutralizing antibodies were not added (–) or (**A**) the EGFR-selective inhibitor AG1478 (10 mcM), an EGFR neutralizing antibody (5 mcg/ml), or the TACE inhibitor TAPI-1 (30 mcM), or (**B**) neutralizing antibodies against IL-1R (10 mcg/ml), IL-1alpha (5 mcg/ml), IL-1beta (5 mcg/ml), or TNFR (10 mcg/ml) were added 30 min before treatment with CFTR-172. **A, B.** Values are means ± SD; n = 5. P<0.05 compared with control (*). P<0.05 compared with CFTR-172 alone (#).

To confirm that CFTR-172-induced IL-8 production was not limited to NCI-H292 cells, we examined NHBE cells. CFTR-172 induced IL-8 production in the NHBE cells markedly (Fig. 5A). Pretreatment with AG1478, with an EGFR neutralizing antibody, or with TAPI-1 prevented the IL-8 production induced by CFTR-172 completely (Fig. 5A), implicating the TACE-EGFR cascade in the IL-8 response in NHBE cells.

In NHBE cells, pretreatment with an IL-1R neutralizing antibody inhibited the IL-8 production induced by CFTR-172 markedly, whereas a TNFR neutralizing antibody had no significant effect on IL-8 production (Fig. 5B), implicating ligand binding to IL-1R in the IL-8 response. Pretreatment with an IL-1alpha neutralizing antibody but not an IL-1beta neutralizing antibody inhibited CFTR-172-induced IL-8 production in the NHBE cells markedly (Fig. 5B), implicating IL-1alpha in the IL-8 response. These results indicate that binding of IL-1alpha to IL-1R leads to CFTR-172-induced IL-8 production in the NHBE cells.

### Studies in airway epithelial cells containing mutant CFTR versus wild-type CFTR

#### The lack of normal CFTR exaggerates IL-8 production in IB3 cells compared to C38 cells TACE- and EGFR-dependently

**Figure 6:**
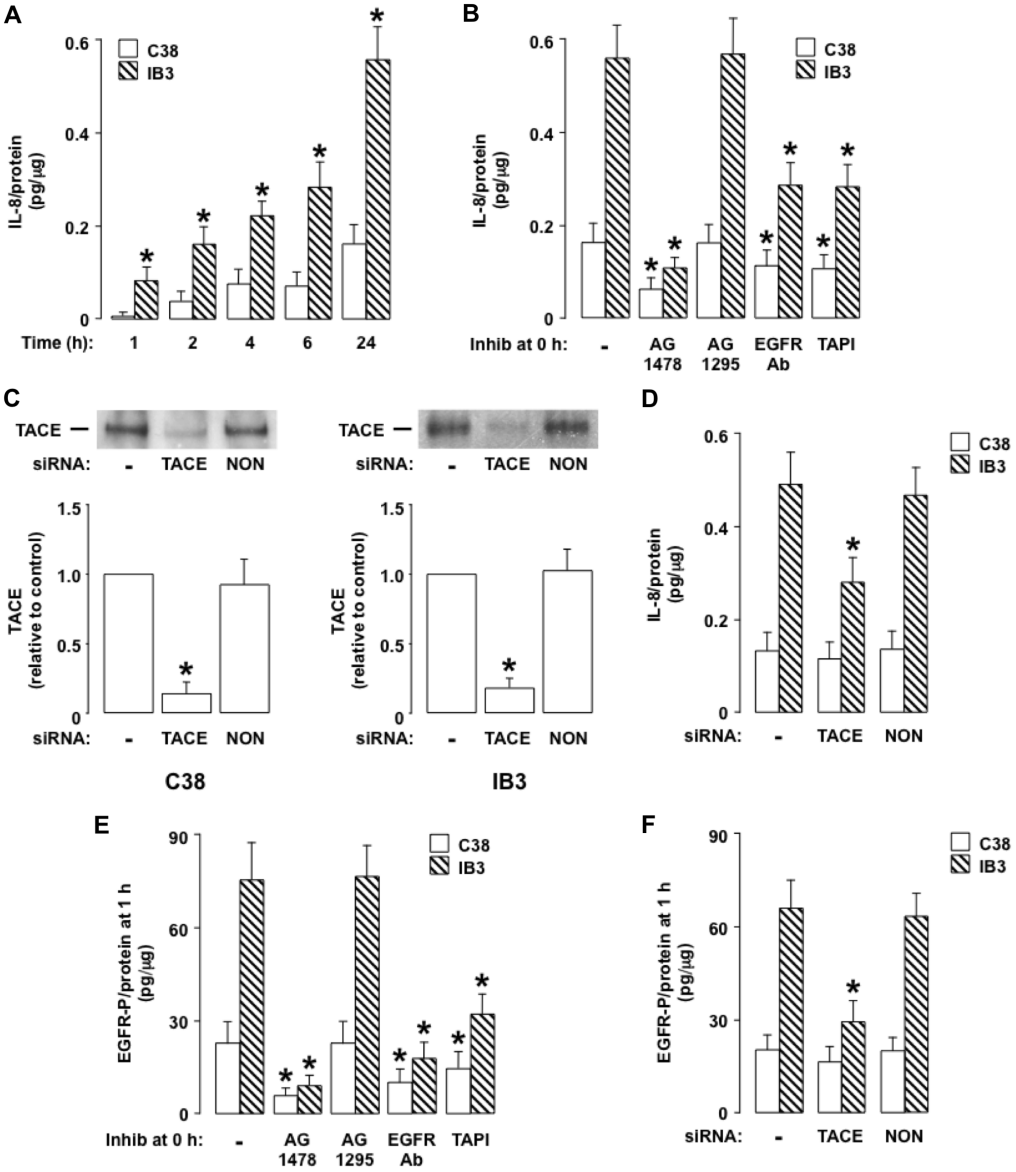
IB3 cells produce more IL-8 than C38 cells EGFR- and TACE-dependently. **A.** IL-8 was measured in C38 cells (open bars) and in IB3 cells (hatched bars) incubated with medium alone for the times indicated. **B, E.** IL-8 (**B**) or EGFR-P (**E**) was measured in C38 cells (open bars) and in IB3 cells (hatched bars) incubated for 24 h (**B**) or 1 h (**E**) with medium alone (–; control). Inhibitors were not added (–) or the EGFR-selective inhibitor AG1478 (10 mcM), the PDGFR-selective inhibitor AG1295 (10 mcM), an EGFR neutralizing antibody (5 mcg/ml), or the TACE inhibitor TAPI-1 (30 mcM) was added at time 0 h. **C.** TACE was measured by immunoblot in C38 cells (left panel) and in IB3 cells (right panel) treated with Lipofectamine 2000 alone (–; control) or transfected with TACE siRNA (100 nM) or non-targeting siRNA (100 nM; NON). A blot representative of three independent experiments is shown. Band intensities were quantified, and the intensity of the control band was set arbitrarily to 1. **D, F.** IL-8 (**D**) or EGFR-P (**F**) was measured in C38 cells (open bars) and in IB3 cells (hatched bars) treated with Lipofectamine 2000 alone (–; control) or transfected with TACE siRNA (100 nM) or nontargeting siRNA (100 nM; NON) and incubated for 72 h before the addition of medium for 24 h (**D**) or 1 h (**F**). **A-F.** Values are means ± SD; n = 5. P<0.05 compared with the C38 cells (*; **A**) or compared with control (*; **B-F**).

Based on the results in airway epithelial cells containing normal CFTR treated with CFTR-172, we hypothesized that an IL-1R-TACE-EGFR pathway could exaggerate IL-8 production in CF airway epithelial cells. To test this hypothesis, we examined constitutive IL-8 production in CF airway epithelial (IB3) cells, which express mutant CFTR [33], and in isogenic C38 cells, which are IB3 cells corrected with wild-type CFTR [34]. The IB3 cells produced markedly more IL-8 at all time points examined (1, 2, 4, 6, and 24 h) than the C38 cells (Fig. 6A), indicating that the IB3 cells produce IL-8 constitutively and in excess of the C38 cells, similar to previous reports [15], [16]. Pretreatment with AG1478 inhibited IL-8 production in the IB3 and C38 cells completely (Fig. 6B), whereas pretreatment with AG1295 had no significant effect on IL-8 production in either cell type (Fig. 6B), implicating EGFR activation in the exaggerated IL-8 production in the IB3 cells.

We hypothesized that removal of CFTR from the epithelial surface activates TACE, resulting in cleavage of EGFR pro-ligands, which then bind to and activate EGFR, causing IL-8 production. Consistent with this idea, in the IB3 cells, pretreatment with an EGFR neutralizing antibody inhibited approximately one-half of the IL-8 response (Fig. 6B). The TACE inhibitor TAPI-1 similarly inhibited IL-8 production in the IB3 cells (Fig. 6B). TACE siRNA knockdown, which markedly decreased TACE protein in IB3 cells and in C38 cells (Fig. 6C), reproduced the inhibitory effects of TAPI on IL-8 production in the IB3 cells (Fig. 6D), confirming that TACE was involved in the exaggerated IL-8 response in the IB3 cells. In contrast, in the C38 cells, an EGFR neutralizing antibody or TAPI-1 decreased IL-8 production only slightly (Fig. 6B), and TACE siRNA knockdown had no significant effect on IL-8 production (Fig. 6D).

Next we examined EGFR-P in the IB3 and C38 cells. In the steady state, the IB3 cells contained approximately 3.2-fold more EGFR-P than the C38 cells (Fig. 6E), and pretreatment with AG1478 decreased EGFR-P to very low levels in both cell types (Fig. 6E). In the IB3 cells, an EGFR neutralizing antibody (Fig. 6E), TAPI-1 (Fig. 6E), or TACE siRNA knockdown (Fig. 6F) inhibited EGFR-P markedly, whereas, in the C38 cells, these treatments inhibited EGFR-P only slightly (Figs 6E, 6F). Together, these results suggest that greater TACE-dependent EGFR-P in the IB3 cells than in the C38 cells exaggerates IL-8 production in the IB3 cells.

Notably, when added to IB3 cells, the CFTR inhibitor CFTR-172 (20 mcM) did not increase IL-8 production at 24 h significantly relative to control (0.61 ± 0.10 vs. 0.56 ± 0.07 pg IL-8/mcg protein; p>0.05; n = 3), suggesting that CFTR-172-induced IL-8 production in NCI-H292 cells (Fig. 1A) and NHBE cells (Fig. 5A) occurs via CFTR inhibition and not via off-target effects.

#### IL-1alpha binding to IL-1R exaggerates EGFR-dependent IL-8 production in IB3 cells but not in C38 cells

**Figure 7:**
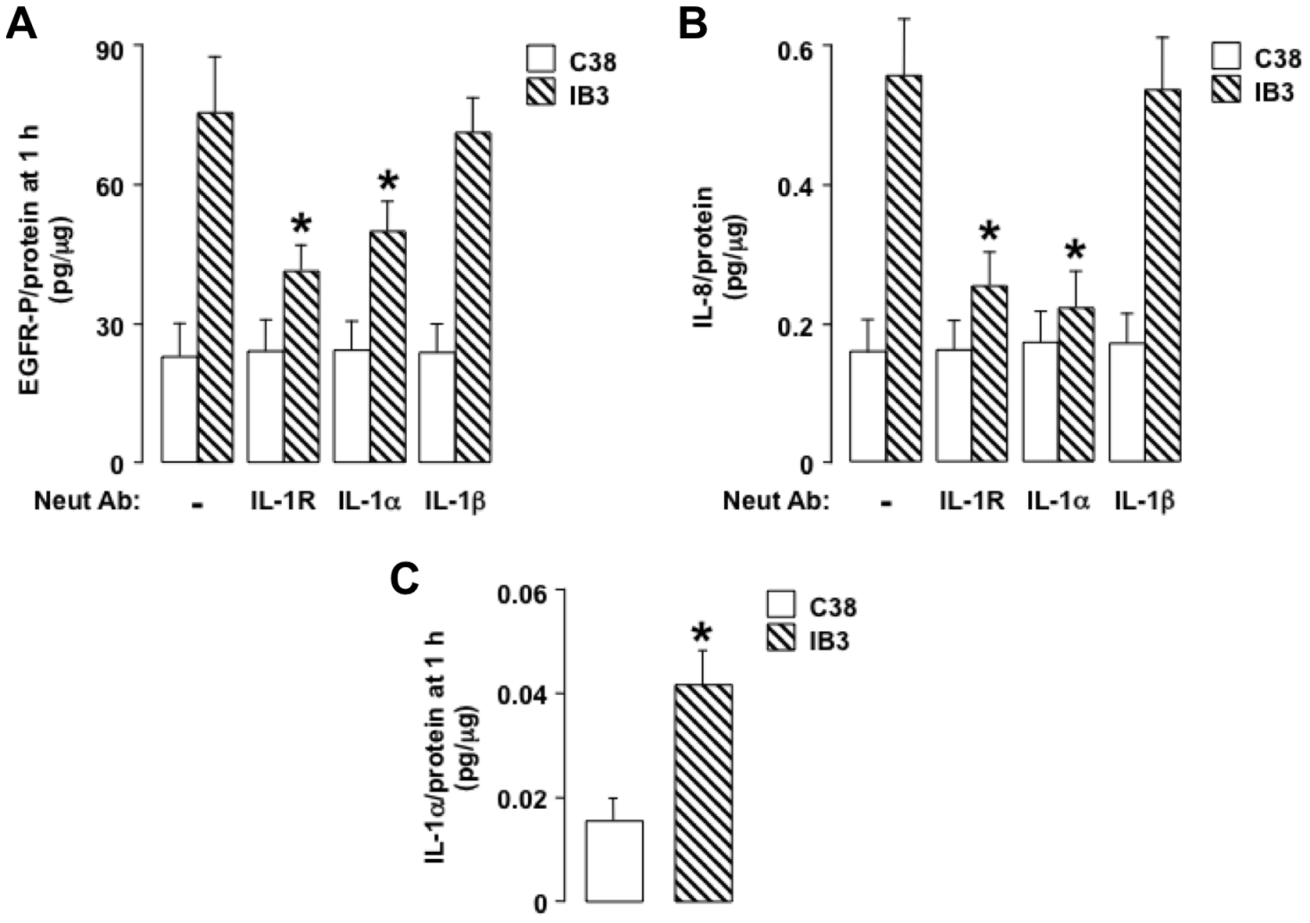
IL-1alpha binding to IL-1R increases EGFR-P and IL-8 production in IB3 cells but not in C38 cells. **A, B.** EGFR-P (**A**) or IL-8 (**B**) was measured in C38 cells (open bars) and in IB3 cells (hatched bars) incubated for 1 h (**A**) or 24 h (**B**) with medium alone (–; control). Neutralizing antibodies were not added (–) or neutralizing antibodies against IL-1R (10 mcg/ml), IL-1alpha (5 mcg/ml), or IL-1beta (5 mcg/ml) were added at time 0 h. **C.** IL-1alpha was measured in cell supernatants of C38 cells (open bar) and IB3 cells (hatched bar) incubated for 1 h with medium alone. **A-C.** Values are means ± SD; n = 5. P<0.05 compared with control (*; **A, B**) or compared with the C38 cells (*; **C**).

Because IL-1R signaling is known to activate the TACE-EGFR cascade [42], [43] and to stimulate IL-8 production [44], we reasoned that exaggerated IL-8 production in CF airway epithelial cells could occur via IL-1R-dependent activation of the TACE-EGFR cascade. Consistent with this idea, in the IB3 cells, pretreatment with an IL-1R neutralizing antibody decreased EGFR-P (Fig. 7A) and IL-8 production (Fig. 7B) markedly, whereas, in the C38 cells, IL-1R blockade had no significant effect on these responses (Figs. 7A, 7B). An IL-1alpha neutralizing antibody had a similar suppressive effect on EGFR-P (Fig. 7A) and on IL-8 production (Fig. 7B) in the IB3 cells but had no significant effect on these responses in the C38 cells (Figs. 7A, 7B). An IL-1beta neutralizing antibody had no effect on EGFR-P (Fig. 7A) or on IL-8 production (Fig. 7B) in either cell type. In addition, there was markedly more IL-1alpha in the supernatants of IB3 cells than of C38 cells (Fig. 7C). Together, these results suggest that binding of IL-1alpha to IL-1R exaggerates TACE-dependent EGFR-P and subsequent IL-8 production in the IB3 cells but not in the C38 cells.

## Discussion

Exuberant airway neutrophilic inflammation is a serious and presently untreated feature of CF airway disease [7]. There is growing evidence that exaggerated production of the neutrophil chemokine IL-8 may be an intrinsic property of airway epithelial cells lacking normal CFTR function [8]–[19], [35], suggesting that normal CFTR may suppress signals leading to IL-8 production. Because EGFR activation induces IL-8 production [20]–[22], in the present study we examined the novel hypothesis that loss of normal CFTR function exaggerates airway epithelial IL-8 production via activation of an EGFR signaling cascade. To test this hypothesis, we utilized two complementary approaches. First, we examined the effects of the CFTR-selective inhibitor CFTR-172 [31] on IL-8 production in airway epithelial (NCI-H292, NHBE) cells containing normal CFTR [32]; and second, we examined the effects of CFTR on constitutive IL-8 production in airway epithelial (IB3) cells containing mutant CFTR [33] and in isogenic (C38) cells complemented with wild-type CFTR [34]. We found that CFTR-172 induces IL-8 production in airway epithelial cells containing normal CFTR and that IB3 cells produce markedly more IL-8 than C38 cells in the constitutive state, consistent with previous studies utilizing CFTR-172 [17]–[19] and IB3 cells and C38 cells [15], [16]. Both CFTR-172-induced IL-8 production in airway epithelial cells containing normal CFTR and exaggerated IL-8 production in IB3 cells versus C38 cells were suppressed by the EGFR-selective inhibitor AG1478 and by pretreatment with an EGFR neutralizing antibody (which prevents ligand binding to EGFR), implicating ligand-dependent EGFR activation in the exaggerated IL-8 responses. This is the first study to show that loss of normal CFTR function exaggerates IL-8 production via activation of an EGFR cascade.

Metalloproteases cleave EGFR pro-ligands and release soluble ligands, making them available for binding to EGFR [25]. Among the metalloproteases known to cleave EGFR pro-ligands on the airway epithelial surface is TACE [26]. In NCI-H292 cells, TAPI-1, a metalloprotease inhibitor with relative selectivity for TACE, and TACE siRNA knockdown prevented EGFR activation and IL-8 production induced by CFTR-172 completely. In NHBE cells, TAPI-1 also prevented EGFR-dependent IL-8 production induced by CFTR-172, implicating TACE-dependent EGFR pro-ligand cleavage in CFTR-172-induced IL-8 production in multiple cell types. Notably, in response to CFTR-172, NCI-H292 cells produced markedly more IL-8 than NHBE cells. The reason for this difference is unknown but may involve the presence of feedback mechanisms that activate the TACE-EGFR-IL-8 cascade in NCI-H292 cells but not in NHBE cells [30]. Further, in IB3 cells, TAPI-1 and TACE siRNA knockdown suppressed exaggerated EGFR-P and IL-8 production markedly, whereas in C38 cells, the effects of TACE inhibition on these responses were minimal, indicating that TACE-dependent EGFR activation contributed to the exuberant IL-8 response in IB3 cells. EGFR and EGFR pro-ligands have been shown to be increased in the airways of subjects with CF [24], [45], suggesting that autocrine or paracrine activation of a surface EGFR cascade occurs in CF. Consistent with this idea, the present findings indicate that loss of normal CFTR function exaggerates IL-8 production via TACE-dependent EGFR activation.

How does the absence (or removal) of normal CFTR function activate a TACE-EGFR-IL-8 cascade? To address this question, we examined NCI-H292 cells treated with CFTR-172 because of the robust IL-8 response in these cells and because the addition of CFTR-172 at the beginning of the experiment enabled us to examine signaling over time. We found that the production and release of IL-8 in response to CFTR-172 is delayed, occurring between 12 and 16 h after treatment. Because direct activation of the EGFR by its ligand TGF-alpha led to IL-8 production and release between 2 and 4 h after stimulation (data not shown), the relative delay in CFTR-172-induced IL-8 release suggests that the EGFR-P leading to the IL-8 response is similarly delayed. Indeed, we found that CFTR-172 induces a late TACE-dependent phase of EGFR-P peaking at 12 h, and we found that this EGFR-P is prevented by pretreatment with cycloheximide, implicating protein synthesis in the late phase of EGFR-P. In other studies, CFTR-172 has been reported to increase reactive oxygen species [46], to increase mitogen-activated protein kinase activity [46], and to increase nuclear factor-kappa B activity [18], [19], raising the possibility that such signals may be involved upstream of the late phase of EGFR-P induced by CFTR-172. Characterizing early signals downstream of CFTR-172 will be an important subject of future studies. Interestingly, Perez et al. reported that IL-8 production in NHBE cells is increased by five days of treatment with CFTR-172 [17], suggesting that a long delay between the addition of CFTR-172 and IL-8 production may also have been present in their study.

**Figure 8:**
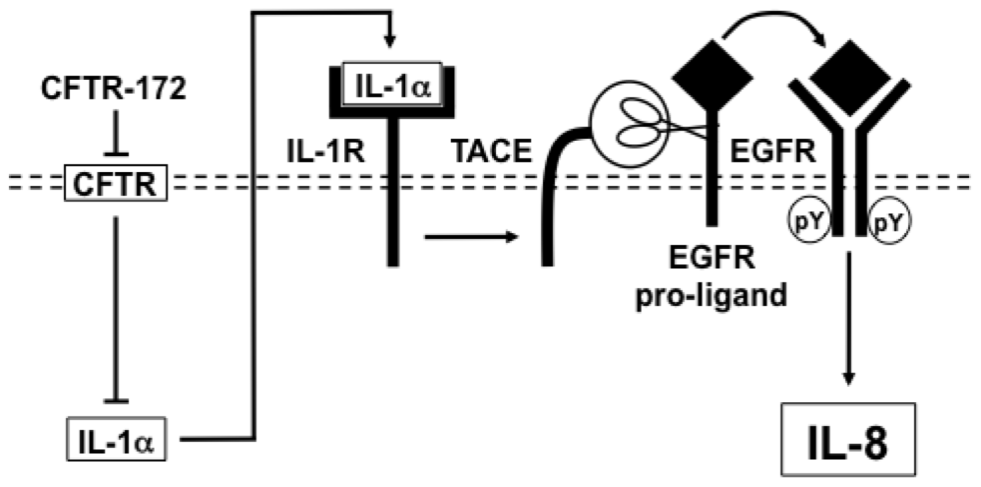
Schematic of the pro-inflammatory IL-1alpha-IL-1R-TACE-EGFR-IL-8 cascade in airway epithelial (NCI-H292) cells treated with a CFTR inhibitor. The CFTR-selective inhibitor CFTR-172 suppresses normal CFTR function, inducing the production and release of IL-1alpha. Binding of IL-1alpha to IL-1R leads to the metalloprotease TACE (scissors)-dependent cleavage of EGFR pro-ligands and subsequent binding of mature EGFR ligand to EGFR (curved solid arrow), resulting in EGFR-P (pY) and in downstream IL-8 production. Similarly, in NHBE cells treated with CFTR-172, and in CF (IB3) cells containing mutant CFTR but not in isogenic C38 cells corrected with wild-type CFTR, exaggerated IL-8 production occurs via IL-1alpha-IL-1R-dependent activation of the TACE-EGFR cascade.

Multiple endogenous epithelial cell products have been shown to activate the TACE-EGFR cascade [30], [47]. We focused on IL-1 for several reasons: First, IL-1 is a well known stimulus of IL-8 production [44]; second, binding of IL-1 to IL-1R activates the TACE-EGFR cascade [42], [43]; third, proteins in the IL-1R signaling pathway are upregulated in CF airway epithelial cells [15], [41]; and fourth, IL-1R blockade has been previously reported to suppress exaggerated IL-8 production in CF airway epithelial cells [41]. A role for EGFR in IL-1R-dependent IL-8 production was not examined in that study. In the present study, we found that, in NCI-H292 cells, neutralizing antibodies against IL-1R or its ligand IL-1alpha, but not its ligand IL-1beta, markedly suppress EGFR-P and IL-8 production induced by CFTR-172. In NHBE cells, blockade of IL-1R or IL-1alpha, but not of IL-1beta, also suppressed CFTR-172-induced IL-8 production, implicating binding of IL-1alpha to IL-1R in IL-8 production induced by CFTR-172 in multiple cell types. In IB3 cells, blockade of IL-1R or IL-1alpha, but not of IL-1beta, markedly suppressed exaggerated EGFR-P and IL-8 production, whereas in C38 cells, blockade of IL-1R or its ligands had no effect on these responses. In NCI-H292 cells, CFTR-172 induced the release of IL-1alpha between 6 and 12 h after treatment, temporally consistent with binding of IL-1alpha to IL-1R contributing to the late phase of EGFR-P at 12 h and to downstream IL-8 production. Further, in IB3 cells, constitutive IL-1alpha levels were higher than in C38 cells. These findings strongly suggest that loss of normal CFTR function exaggerates IL-8 production via activation of an IL-1alpha IL-1R TACE EGFR cascade and that the presence of normal CFTR function suppresses this cascade (shown as a schematic in Fig. 8).

Notably, blockade of IL-1alpha, but not of IL-1beta, suppressed IL-8 production in airway epithelial (NCI-H292, NHBE) cells treated with CFTR-172 and in IB3 (CF) cells. In NCI-H292 cells, the addition of exogenous IL-1beta (20 ng/ml) markedly increased IL-8 levels at 4 h TACE- and EGFR-dependently (data not shown), indicating that binding of IL-1beta to IL-1R activates the TACE-EGFR-IL-8 cascade. This result suggests that IL-1beta may not be released in response to CFTR blockade. Indeed, unlike IL-1alpha, we could not find IL-1beta in the supernatants of NCI-H292 cells treated with CFTR-172 or in the supernatants of IB3 cells in the constitutive state (lower limit of detection approximately 4 pg/ml), similar to a recent study that reported no IL-1beta production in CF airway epithelial cells in the constitutive state or after exposure to *Pseudomonas* products [48]. Olaru and Jensen reported that 10-fold more IL-1alpha than IL-1beta is produced in human keratinocytes stimulated with Toll-like receptor (TLR)-2 ligands [49], suggesting that greater production of IL-1alpha than IL-1beta may be a common feature of epithelial cells. These authors also reported that TLR-2-dependent IL-8 production occurs via an autocrine cascade involving IL-1alpha but not IL-1beta [49]. Previously, we showed that TLR-2-dependent IL-8 production occurs via activation of the TACE-EGFR cascade [29], leading us to speculate that IL-1alpha binding to IL-1R may activate the TACE-EGFR-IL-8 cascade downstream of TLR-2. Further, blockade of IL-1alpha, but not of IL-1beta, has been reported to suppress airway neutrophilic inflammation induced by cigarette smoke in mice [50], and to suppress production of an IL-8 homolog in rat alveolar epithelial cells [51]. Thus, there is mounting evidence that IL-1alpha is an important epithelial-derived stimulus for IL-8 production.

There is controversy over whether exaggerated IL-8 production is an intrinsic property of airway epithelial cells lacking normal CFTR [18], [52] or whether it is the result of impaired mucociliary clearance [53] and bacterial infection [54]. Our study was not designed to address this issue but rather to test the idea that loss of normal CFTR activates an airway epithelial TACE-EGFR-IL-8 signaling pathway in available model systems of CFTR deficiency. We chose the CFTR inhibitor CFTR-172 because CFTR-172 has been shown to induce IL-8 production in airway epithelial cells containing normal CFTR [17]–[19]. Similarly, IB3 cells containing mutant CFTR have been consistently reported to produce more IL-8 than isogenic C38 cells complemented with wild-type CFTR [10], [12], [15], [16], [35]–[37]. However, these model systems have limitations. CFTR-172 has been reported to have possible off-target effects [55] and immortalized CF cell lines are subject to clonal variation and genetic drift [52]. This may explain why some immortalized CF airway cells have been reported to produce equal, or even lesser, amounts of IL-8 compared to wild-type CFTR-complemented cells after exposure to bacterial products, viral products, or in the constitutive state [56]–[60]. Thus, there is serious concern that immortalized CF cells may have little relevance to primary airway tissues from CF subjects.

On the whole, when grown *in vitro*, primary airway epithelial cells from CF subjects have not been reported to produce more IL-8 in the constitutive state or in response to inflammatory stimuli than primary airway cells from subjects without CF [16], [56], [61], [62]. However, under certain culture conditions (eg, early after establishment of air-liquid interface culture [61], in the presence of serum [62], or before introduction of wild-type CFTR [52]), primary CF airway cells have been reported to produce more IL-8 than their non-CF counterparts. The reasons for these variable results are uncertain but could be due to genetic differences not related to CFTR, to epigenetic modifications of the IL-8 gene in primary airway epithelial cells from CF subjects [63], or to differences in cell culture conditions [16]. Clearly, further studies in primary CF versus non-CF airway cells are needed to determine whether pro-inflammatory EGFR signaling is increased in CF cells.

In the present study, we addressed possible off-target effects of CFTR-172 as a potential confounder. In IB3 cells containing mutant CFTR, CFTR-172 did not increase IL-8 production at 24 h significantly, similar to a previous study reporting that CFTR-172 did not increase IL-8 production in immortalized CF airway cells or in primary CF airway cells [17]. Together, these findings suggest that CFTR-172-induced IL-8 production in NCI-H292 cells and NHBE cells occurs via inhibition of wild-type CFTR and not via off-target effects. We also addressed the presence of serum as a potential confounder. We found that longer periods of serum starvation (eg, overnight) decreased the amount of IL-8 produced by NCI-H292 cells treated with CFTR-172 and by IB3 cells in the steady state (data not shown), similar to previous studies reporting that the presence of serum in culture medium increases constitutive IL-8 production in airway epithelial cells containing mutant CFTR but not in isogenic cells containing wild-type CFTR [12], [15], [35]. For this reason, in the present study, NCI-H292 cells and NHBE cells were cultured in serum-free (or EGF-free) medium for only 2 h before CFTR-172 treatment, while responses in IB3 cells and C38 cells were measured beginning only 1 h after the switch from serum-containing medium to serum-free medium. Because serum contains EGFR ligands, and because EGFR ligands have been reported to upregulate expression of EGFR pro-ligands [64], [65] and of EGFR [64], we speculate that serum may increase expression of components of the surface EGFR cascade, which can be subsequently activated by removal of the constraining effects of CFTR.

In summary, we show that the CFTR-selective inhibitor CFTR-172 induces IL-8 production in human airway epithelial (NCI-H292, NHBE) cells containing normal CFTR via metalloprotease TACE-dependent activation of an EGFR cascade. Further, we show that a TACE-EGFR cascade exaggerates constitutive IL-8 production in human airway epithelial (IB3) cells containing mutant CFTR but not in isogenic C38 cells containing wild-type CFTR. Binding of IL-1alpha to its receptor stimulated the TACE-EGFR-IL-8 cascade in airway epithelial (NCI-H292, NHBE) cells treated with CFTR-172 and in IB3 cells, but not in C38 cells, exaggerating IL-8 production only in the airway cells lacking normal CFTR function. Thus, we conclude that normal CFTR suppresses airway epithelial IL-8 production that occurs via a stimulatory EGFR cascade, and that loss of normal CFTR activity exaggerates IL-8 production via activation of a pro-inflammatory EGFR cascade. The present findings suggest that components of this signaling cascade such as IL-1alpha and EGFR could be novel therapeutic targets for exuberant neutrophilic inflammation in CF airways.

## References

[1] Baggiolini M, Walz A, Kunkel SL (1989) Neutrophil-activating peptide-1/interleukin 8, a novel cytokine that activates neutrophils. J Clin Invest 84: 1045–1049. 2677047 10.1172/JCI114265 PMC329758

[2] Kerem B, Rommens JM, Buchanan JA, Markiewicz D, Cox TK, et al (1989) Identification of the cystic fibrosis gene: genetic analysis. Science 245: 1073–1080. 2570460 10.1126/science.2570460

[3] Riordan JR, Rommens JM, Kerem B, Alon N, Rozmahel R, et al (1989) Identification of the cystic fibrosis gene: cloning and characterization of complementary DNA. Science 245: 1066–1073. 2475911 10.1126/science.2475911

[4] Bonfield TL, Panuska JR, Konstan MW, Hilliard KA, Hilliard JB, et al (1995) Inflammatory cytokines in cystic fibrosis lungs. Am J Respir Crit Care Med 152: 2111–2118. 8520783 10.1164/ajrccm.152.6.8520783

[5] Dean TP, Dai Y, Shute JK, Church MK, Warner JO (1993) Interleukin-8 concentrations are elevated in bronchoalveolar lavage, sputum, and sera of children with cystic fibrosis. Pediatr Res 34: 159–161. 8233718 10.1203/00006450-199308000-00010

[6] Salva PS, Doyle NA, Graham L, Eigen H, Doerschuk CM (1996) TNF-alpha, IL-8, soluble ICAM-1, and neutrophils in sputum of cystic fibrosis patients. Pediatr Pulmonol 21: 11–19. 8776260 10.1002/(SICI)1099-0496(199601)21:1<11::AID-PPUL2>3.0.CO;2-T

[7] Davis PB, Drumm M, Konstan MW (1996) Cystic fibrosis. Am J Respir Crit Care Med 154: 1229–1256. 8912731 10.1164/ajrccm.154.5.8912731

[8] Khan TZ, Wagener JS, Bost T, Martinez J, Accurso FJ, et al (1995) Early pulmonary inflammation in infants with cystic fibrosis. Am J Respir Crit Care Med 151: 1075–1082. 7697234 10.1164/ajrccm/151.4.1075

[9] Tirouvanziam R, Khazaal I, Peault B (2002) Primary inflammation in human cystic fibrosis small airways. Am J Physiol Lung Cell Mol Physiol 283: L445–51. 12114207 10.1152/ajplung.00419.2001

[10] DiMango E, Ratner AJ, Bryan R, Tabibi S, Prince A (1998) Activation of NF-kappaB by adherent Pseudomonas aeruginosa in normal and cystic fibrosis respiratory epithelial cells. J Clin Invest 101: 2598–2605. 9616231 10.1172/JCI2865 PMC508849

[11] Kube D, Sontich U, Fletcher D, Davis PB (2001) Proinflammatory cytokine responses to P. aeruginosa infection in human airway epithelial cell lines. Am J Physiol Lung Cell Mol Physiol 280: L493–502. 11159033 10.1152/ajplung.2001.280.3.L493

[12] Li J, Johnson XD, Iazvovskaia S, Tan A, Lin A, et al (2003) Signaling intermediates required for NF-kappa B activation and IL-8 expression in CF bronchial epithelial cells. Am J Physiol Lung Cell Mol Physiol 284: L307–15. 12388360 10.1152/ajplung.00086.2002

[13] Stecenko AA, King G, Torii K, Breyer RM, Dworski R, et al (2001) Dysregulated cytokine production in human cystic fibrosis bronchial epithelial cells. Inflammation 25: 145–155. 11403205 10.1023/a:1011080229374

[14] Tabary O, Zahm JM, Hinnrasky J, Couetil JP, Cornillet P, et al (1998) Selective up-regulation of chemokine IL-8 expression in cystic fibrosis bronchial gland cells in vivo and in vitro. Am J Pathol 153: 921–930. 9736040 10.1016/S0002-9440(10)65633-7 PMC1853001

[15] Eidelman O, Srivastava M, Zhang J, Leighton X, Murtie J, et al (2001) Control of the proinflammatory state in cystic fibrosis lung epithelial cells by genes from the TNF-alphaR/NFkappaB pathway. Mol Med 7: 523–534. 11591888 PMC1950060

[16] Aldallal N, McNaughton EE, Manzel LJ, Richards AM, Zabner J, et al (2002) Inflammatory response in airway epithelial cells isolated from patients with cystic fibrosis. Am J Respir Crit Care Med 166: 1248–1256. 12403695 10.1164/rccm.200206-627OC

[17] Perez A, Issler AC, Cotton CU, Kelley TJ, Verkman AS, et al (2007) CFTR inhibition mimics the cystic fibrosis inflammatory profile. Am J Physiol Lung Cell Mol Physiol 292: L383–95. 16920886 10.1152/ajplung.00403.2005

[18] Vij N, Mazur S, Zeitlin PL (2009) CFTR is a negative regulator of NFkappaB mediated innate immune response. PLoS One 4: e4664. 19247502 10.1371/journal.pone.0004664 PMC2647738

[19] Hunter MJ, Treharne KJ, Winter AK, Cassidy DM, Land S, et al (2010) Expression of wild-type CFTR suppresses NF-kappaB-driven inflammatory signalling. PLoS One 5: e11598. 20644644 10.1371/journal.pone.0011598 PMC2904384

[20] Nakanaga T, Nadel JA, Ueki IF, Koff JL, Shao MX (2007) Regulation of interleukin-8 via an airway epithelial signaling cascade. Am J Physiol Lung Cell Mol Physiol 292: L1289–96. 17220369 10.1152/ajplung.00356.2006

[21] Richter A, O'Donnell RA, Powell RM, Sanders MW, Holgate ST, et al (2002) Autocrine ligands for the epidermal growth factor receptor mediate interleukin-8 release from bronchial epithelial cells in response to cigarette smoke. Am J Respir Cell Mol Biol 27: 85–90. 12091250 10.1165/ajrcmb.27.1.4789

[22] Subauste MC, Proud D (2001) Effects of tumor necrosis factor-alpha, epidermal growth factor and transforming growth factor-alpha on interleukin-8 production by, and human rhinovirus replication in, bronchial epithelial cells. Int Immunopharmacol 1: 1229–1234. 11460304 10.1016/s1567-5769(01)00063-7

[23] Takeyama K, Fahy JV, Nadel JA (2001) Relationship of epidermal growth factor receptors to goblet cell production in human bronchi. Am J Respir Crit Care Med 163: 511–516. 11179132 10.1164/ajrccm.163.2.2001038

[24] Burgel PR, Montani D, Danel C, Dusser DJ, Nadel JA (2007) A morphometric study of mucins and small airway plugging in cystic fibrosis. Thorax 62: 153–161. 16928707 10.1136/thx.2006.062190 PMC2111259

[25] Peschon JJ, Slack JL, Reddy P, Stocking KL, Sunnarborg SW, et al (1998) An essential role for ectodomain shedding in mammalian development. Science 282: 1281–1284. 9812885 10.1126/science.282.5392.1281

[26] Shao MX, Ueki IF, Nadel JA (2003) Tumor necrosis factor alpha-converting enzyme mediates MUC5AC mucin expression in cultured human airway epithelial cells. Proc Natl Acad Sci U S A 100: 11618–11623. 12972643 10.1073/pnas.1534804100 PMC208807

[27] Kuwahara I, Lillehoj EP, Lu W, Singh IS, Isohama Y, et al (2006) Neutrophil elastase induces IL-8 gene transcription and protein release through p38/NF-{kappa}B activation via EGFR transactivation in a lung epithelial cell line. Am J Physiol Lung Cell Mol Physiol 291: L407–16. 16632517 10.1152/ajplung.00471.2005

[28] Liu K, Gualano RC, Hibbs ML, Anderson GP, Bozinovski S (2008) Epidermal growth factor receptor signaling to Erk1/2 and STATs control the intensity of the epithelial inflammatory responses to rhinovirus infection. J Biol Chem 283: 9977–9985. 18276593 10.1074/jbc.M710257200

[29] Koff JL, Shao MX, Ueki IF, Nadel JA (2008) Multiple TLRs activate EGFR via a signaling cascade to produce innate immune responses in airway epithelium. Am J Physiol Lung Cell Mol Physiol 294: L1068–75. 18375743 10.1152/ajplung.00025.2008

[30] Kim S, Lewis C, Nadel JA (2011) Epidermal growth factor receptor reactivation induced by E-prostanoid-3 receptor- and tumor necrosis factor-alpha-converting enzyme-dependent feedback exaggerates interleukin-8 production in airway cancer (NCI-H292) cells. Exp Cell Res 317: 2650–2660. 21925169 10.1016/j.yexcr.2011.08.023

[31] Ma T, Thiagarajah JR, Yang H, Sonawane ND, Folli C, et al (2002) Thiazolidinone CFTR inhibitor identified by high-throughput screening blocks cholera toxin-induced intestinal fluid secretion. J Clin Invest 110: 1651–1658. 12464670 10.1172/JCI16112 PMC151633

[32] Nagayama S, Kai H, Okiyoneda T, Horikawa S, Miyata T (1999) Characterization of CFTR expression in a human pulmonary mucoepidermoid carcinoma cell line, NCI-H292 cells. FEBS Lett 455: 215–218. 10437775 10.1016/s0014-5793(99)00880-7

[33] Zeitlin PL, Lu L, Rhim J, Cutting G, Stetten G, et al (1991) A cystic fibrosis bronchial epithelial cell line: immortalization by adeno-12-SV40 infection. Am J Respir Cell Mol Biol 4: 313–319. 1849726 10.1165/ajrcmb/4.4.313

[34] Flotte TR, Afione SA, Solow R, Drumm ML, Markakis D, et al (1993) Expression of the cystic fibrosis transmembrane conductance regulator from a novel adeno-associated virus promoter. J Biol Chem 268: 3781–3790. 7679117

[35] Venkatakrishnan A, Stecenko AA, King G, Blackwell TR, Brigham KL, et al (2000) Exaggerated activation of nuclear factor-kappaB and altered IkappaB-beta processing in cystic fibrosis bronchial epithelial cells. Am J Respir Cell Mol Biol 23: 396–403. 10970832 10.1165/ajrcmb.23.3.3949

[36] Bezzerri V, Borgatti M, Finotti A, Tamanini A, Gambari R, et al (2011) Mapping the transcriptional machinery of the IL-8 gene in human bronchial epithelial cells. J Immunol 187: 6069–6081. 22031759 10.4049/jimmunol.1100821

[37] Bhattacharyya S, Balakathiresan NS, Dalgard C, Gutti U, Armistead D, et al (2011) Elevated miR-155 promotes inflammation in cystic fibrosis by driving hyperexpression of interleukin-8. J Biol Chem 286: 11604–11615. 21282106 10.1074/jbc.M110.198390 PMC3064214

[38] Banks-Schlegel SP, Gazdar AF, Harris CC (1985) Intermediate filament and cross-linked envelope expression in human lung tumor cell lines. Cancer Res 45: 1187–1197. 2578876

[39] Wong J, Korcheva V, Jacoby DB, Magun BE (2007) Proinflammatory responses of human airway cells to ricin involve stress-activated protein kinases and NF-kappaB. Am J Physiol Lung Cell Mol Physiol 293: L1385–94. 17873006 10.1152/ajplung.00207.2007

[40] Zhang Z, Oliver P, Lancaster JJ, Schwarzenberger PO, Joshi MS, et al (2001) Reactive oxygen species mediate tumor necrosis factor alpha-converting, enzyme-dependent ectodomain shedding induced by phorbol myristate acetate. FASEB J 15: 303–305. 11156944 10.1096/fj.00-0371fje

[41] Verhaeghe C, Remouchamps C, Hennuy B, Vanderplasschen A, Chariot A, et al (2007) Role of IKK and ERK pathways in intrinsic inflammation of cystic fibrosis airways. Biochem Pharmacol 73: 1982–1994. 17466952 10.1016/j.bcp.2007.03.019

[42] Xu P, Derynck R (2010) Direct activation of TACE-mediated ectodomain shedding by p38 MAP kinase regulates EGF receptor-dependent cell proliferation. Mol Cell 37: 551–566. 20188673 10.1016/j.molcel.2010.01.034 PMC4240279

[43] Hall KC, Blobel CP (2012) Interleukin-1 stimulates ADAM17 through a mechanism independent of its cytoplasmic domain or phosphorylation at threonine 735. PLoS One 7: e31600. 22384041 10.1371/journal.pone.0031600 PMC3288042

[44] Strieter RM, Kunkel SL, Showell HJ, Remick DG, Phan SH, et al (1989) Endothelial cell gene expression of a neutrophil chemotactic factor by TNF-alpha, LPS, and IL-1 beta. Science 243: 1467–1469. 2648570 10.1126/science.2648570

[45] Hardie WD, Bejarano PA, Miller MA, Yankaskas JR, Ritter JH, et al (1999) Immunolocalization of transforming growth factor alpha and epidermal growth factor receptor in lungs of patients with cystic fibrosis. Pediatr Dev Pathol 2: 415–423. 10441618 10.1007/s100249900144

[46] Maiuri L, Luciani A, Giardino I, Raia V, Villella VR, et al (2008) Tissue transglutaminase activation modulates inflammation in cystic fibrosis via PPARgamma down-regulation. J Immunol 180: 7697–7705. 18490773 10.4049/jimmunol.180.11.7697

[47] Kim S, Lewis C, Nadel JA (2011) CCL20/CCR6 feedback exaggerates epidermal growth factor receptor-dependent MUC5AC mucin production in human airway epithelial (NCI-H292) cells. J Immunol 186: 3392–3400. 21300824 10.4049/jimmunol.1003377

[48] Tang A, Sharma A, Jen R, Hirschfeld AF, Chilvers MA, et al (2012) Inflammasome-mediated IL-1beta production in humans with cystic fibrosis. PLoS One 7: e37689. 22649552 10.1371/journal.pone.0037689 PMC3359311

[49] Olaru F, Jensen LE (2010) Staphylococcus aureus stimulates neutrophil targeting chemokine expression in keratinocytes through an autocrine IL-1alpha signaling loop. J Invest Dermatol 130: 1866–1876. 20182449 10.1038/jid.2010.37 PMC2886182

[50] Botelho FM, Bauer CM, Finch D, Nikota JK, Zavitz CC, et al (2011) IL-1alpha/IL-1R1 expression in chronic obstructive pulmonary disease and mechanistic relevance to smoke-induced neutrophilia in mice. PLoS One 6: e28457. 22163019 10.1371/journal.pone.0028457 PMC3232226

[51] Manzer R, Dinarello CA, McConville G, Mason RJ (2008) Ozone exposure of macrophages induces an alveolar epithelial chemokine response through IL-1alpha. Am J Respir Cell Mol Biol 38: 318–323. 17901407 10.1165/rcmb.2007-0250OC PMC2258451

[52] Veit G, Bossard F, Goepp J, Verkman AS, Galietta LJ, et al (2012) Proinflammatory cytokine secretion is suppressed by TMEM16A or CFTR channel activity in human cystic fibrosis bronchial epithelia. Mol Biol Cell 23: 4188–4202. 22973054 10.1091/mbc.E12-06-0424 PMC3484098

[53] Matsui H, Grubb BR, Tarran R, Randell SH, Gatzy JT, et al (1998) Evidence for periciliary liquid layer depletion, not abnormal ion composition, in the pathogenesis of cystic fibrosis airways disease. Cell 95: 1005–1015. 9875854 10.1016/s0092-8674(00)81724-9

[54] Stoltz DA, Meyerholz DK, Pezzulo AA, Ramachandran S, Rogan MP, et al (2010) Cystic fibrosis pigs develop lung disease and exhibit defective bacterial eradication at birth. Sci Transl Med 2: 29ra31. 10.1126/scitranslmed.3000928 PMC2889616 20427821

[55] Kelly M, Trudel S, Brouillard F, Bouillaud F, Colas J, et al (2010) Cystic fibrosis transmembrane regulator inhibitors CFTR(inh)-172 and GlyH-101 target mitochondrial functions, independently of chloride channel inhibition. J Pharmacol Exp Ther 333: 60–69. 20051483 10.1124/jpet.109.162032

[56] Hybiske K, Fu Z, Schwarzer C, Tseng J, Do J, et al (2007) Effects of cystic fibrosis transmembrane conductance regulator and DeltaF508CFTR on inflammatory response, ER stress, and Ca2+ of airway epithelia. Am J Physiol Lung Cell Mol Physiol 293: L1250–60. 17827250 10.1152/ajplung.00231.2007

[57] Reiniger N, Ichikawa JK, Pier GB (2005) Influence of cystic fibrosis transmembrane conductance regulator on gene expression in response to Pseudomonas aeruginosa infection of human bronchial epithelial cells. Infect Immun 73: 6822–6830. 16177360 10.1128/IAI.73.10.6822-6830.2005 PMC1230967

[58] John G, Yildirim AO, Rubin BK, Gruenert DC, Henke MO (2010) TLR-4-mediated innate immunity is reduced in cystic fibrosis airway cells. Am J Respir Cell Mol Biol 42: 424–431. 19502387 10.1165/rcmb.2008-0408OC PMC5459530

[59] Massengale AR, Quinn FJ, Yankaskas J, Weissman D, McClellan WT, et al (1999) Reduced interleukin-8 production by cystic fibrosis airway epithelial cells. Am J Respir Cell Mol Biol 20: 1073–1080. 10226079 10.1165/ajrcmb.20.5.3243

[60] Black HR, Yankaskas JR, Johnson LG, Noah TL (1998) Interleukin-8 production by cystic fibrosis nasal epithelial cells after tumor necrosis factor-alpha and respiratory syncytial virus stimulation. Am J Respir Cell Mol Biol 19: 210–215. 9698592 10.1165/ajrcmb.19.2.3053

[61] Ribeiro CM, Paradiso AM, Schwab U, Perez-Vilar J, Jones L, et al (2005) Chronic airway infection/inflammation induces a Ca2+i-dependent hyperinflammatory response in human cystic fibrosis airway epithelia. J Biol Chem 280: 17798–17806. 15746099 10.1074/jbc.M410618200

[62] Becker MN, Sauer MS, Muhlebach MS, Hirsh AJ, Wu Q, et al (2004) Cytokine secretion by cystic fibrosis airway epithelial cells. Am J Respir Crit Care Med 169: 645–653. 14670800 10.1164/rccm.200207-765OC

[63] Bartling TR, Drumm ML (2009) Loss of CFTR results in reduction of histone deacetylase 2 in airway epithelial cells. Am J Physiol Lung Cell Mol Physiol 297: L35–43. 19411311 10.1152/ajplung.90399.2008 PMC2711807

[64] Bjorge JD, Paterson AJ, Kudlow JE (1989) Phorbol ester or epidermal growth factor (EGF) stimulates the concurrent accumulation of mRNA for the EGF receptor and its ligand transforming growth factor-alpha in a breast cancer cell line. J Biol Chem 264: 4021–4027. 2521857

[65] Coffey RJJ, Derynck R, Wilcox JN, Bringman TS, Goustin AS, et al (1987) Production and auto-induction of transforming growth factor-alpha in human keratinocytes. Nature 328: 817–820. 2442615 10.1038/328817a0

